# Post-bariatric surgery nutritional follow-up in primary care: a population-based cohort study

**DOI:** 10.3399/bjgp20X714161

**Published:** 2021-04-07

**Authors:** Helen M Parretti, Anuradhaa Subramanian, Nicola J Adderley, Sally Abbott, Abd A Tahrani, Krishnarajah Nirantharakumar

**Affiliations:** Norwich Medical School, Faculty of Medicine and Health, University of East Anglia, Norwich.; Institute of Applied Health Research, University of Birmingham, Birmingham.; Institute of Applied Health Research, University of Birmingham, Birmingham.; Institute of Metabolism and Systems Research, University of Birmingham, Birmingham; Department of Diabetes, Endocrinology and Weight Management, University Hospitals Birmingham NHS Foundation Trust, Birmingham.; Institute of Metabolism and Systems Research, University of Birmingham, Birmingham; Centre for Endocrinology Diabetes and Metabolism (CEDAM), Birmingham Health Partners, Birmingham; Department of Diabetes, Endocrinology and Weight Management, University Hospitals Birmingham NHS Foundation Trust, Birmingham.; Institute of Applied Health Research, University of Birmingham, Birmingham; CEDAM, Birmingham Health Partners, Birmingham; Department of Diabetes, Endocrinology and Weight Management, University Hospitals Birmingham NHS Foundation Trust, Birmingham; Midlands Health Data Research UK.

**Keywords:** bariatric surgery, cohort studies, followup, general practice, nutrition, The Health Improvement Network

## Abstract

**Background:**

Bariatric surgery is the most effective treatment for severe obesity. However, without recommended follow-up it has long-term risks.

**Aim:**

To investigate whether nutritional and weight monitoring in primary care meets current clinical guidance, after patients are discharged from specialist bariatric care.

**Design and setting:**

Retrospective cohort study in primary care practices contributing to IQVIA Medical Research Data in the UK (1 January 2000 to 17 January 2018).

**Method:**

Participants were adults who had had bariatric surgery with a minimum of 3 years’ follow-up post-surgery, as this study focused on patients discharged from specialist care (at 2 years post-surgery). Outcomes were the annual proportion of patients from 2 years post-surgery with a record of recommended nutritional screening blood tests, weight measurement, and prescription of nutritional supplements, and the proportions with nutritional deficiencies based on blood tests.

**Results:**

A total of 3137 participants were included in the study, and median follow-up post-surgery was 5.7 (4.2–7.6) years. Between 45% and 59% of these patients had an annual weight measurement. The greatest proportions of patients with a record of annual nutritional blood tests were for tests routinely conducted in primary care, for example, recorded haemoglobin measurement varied between 44.9% (*n* = 629/1400) and 61.2% (*n* = 653/1067). Annual proportions of blood tests specific to bariatric surgery were low, for example, recorded copper measurement varied between 1.2% (*n* = 10/818) and 1.5% (*n* = 16/1067) where recommended. Results indicated that the most common deficiency was anaemia. Annual proportions of patients with prescriptions for recommended nutritional supplements were low.

**Conclusion:**

This study suggests that patients who have bariatric surgery are not receiving the recommended nutritional monitoring after discharge from specialist care. GPs and patients should be supported to engage with follow-up care. Future research should aim to understand the reasons underpinning these findings.

## INTRODUCTION

Obesity is a healthcare priority, with overweight- and obesity-related ill health estimated to cost the NHS £6.1 billion/year.^1^^,^^2^ Bariatric surgery is recognised as the most clinically and cost-effective treatment for severe and complex obesity.^3^^,^^4^ Globally, the annual rate of bariatric surgery procedures is increasing, leading to a growing cohort of patients living with a history of bariatric surgery.^5^ Bariatric surgery is associated with multiple health benefits such as remission of type 2 diabetes mellitus, improvements in cardiovascular disease, and reduction in all-cause mortality.^6^^,^^7^ However, despite these benefits, without adequate follow-up, bariatric surgery has long-term risks including significant nutritional deficiencies and weight regain, and, for some, the consequences can be significant.^8^^–^^10^ For example, there have been case reports of nutritional deficiencies leading to night-blindness, cardiomyopathy, and neuropathy, including permanent disability or death in some cases.^11^^–^^15^ These case reports commonly cite inadequate follow-up or adherence to supplements as a contributing factor. There is also evidence from cohort studies and systematic reviews that poor follow-up care and adherence to supplements have negative impacts on outcomes.^16^^–^^18^

The importance of follow-up care is recognised in clinical guidance. In the UK, the National Institute for Health and Care Excellence (NICE)^3^^,^^19^ recommends that patients stay under specialist surgical care for the first 2 years post-bariatric surgery, and are then discharged to primary care for annual reviews under a shared-care model with a bariatric specialist. NICE also recommends that annual reviews include nutritional monitoring as a minimum, but does not give any detailed guidance as to what constitutes an adequate nutritional review.^3^ The European Association for the Study of Obesity (EASO)^5^ has also published guidance on post-bariatric surgery management, which highlights the need for long-term follow-up and includes recommendations on monitoring and supplementation. In the UK, the British Obesity and Metabolic Surgery Society (BOMSS) nutritional guidelines are the most detailed clinical guidance available for nutritional monitoring and supplementation post-bariatric surgery^20^ (Box 1).

**Table:** How this fits in

Post-bariatric surgery clinical guidelines recommend lifelong annual nutritional and weight monitoring under a shared-care model between primary care and bariatric specialists. Lack of post-bariatric surgery follow-up can lead to poorer outcomes and detrimental health impacts. The results of this study suggest that most patients who have had bariatric surgery do not receive the recommended annual nutritional reviews or weight monitoring in general practice. There is an urgent need to support GPs and patients to undertake these reviews and to investigate the findings further to improve outcomes and patient safety.

**Box 1.** Abbreviated summary of BOMSS post-surgery nutritional guidance for blood tests and supplements^20^

**Tests/supplements**	**Annual screening blood tests**			**Nutritional supplementation**		
	**LAGB**	**Gastric bypass**	**Sleeve gastrectomy**	**LAGB**	**Gastric bypass**	**Sleeve gastrectomy**
FBC	**x**	**x**	**x**			
U&Es	**x**	**x**	**x**			
LFTs	**x**	**x**	**x**			
Ferritin		**x**	**x**			
Folate		**x**	**x**			
Calcium		**x**	**x**			
Vitamin D		**x**	**x**			
PTH		**x**	**x**			
Thiamine		**S**	**S**			
Vitamin B12		**x**	**x**			
Zinc		**x**				
Copper		**x**				
Vitamin A		**S**				
Vitamin E		**S**				
Vitamin K		**S**				
Selenium		**S**				
Multivitamin supplement				**x**	**x**	**x**
Iron supplement					**x**	**x**
Folic acid supplement					**x**	**x**
Vitamin B12 supplement					**x**	**x**
Calcium and vitamin D supplement					**x**	**x**

*BOMSS = British Obesity and Metabolic Surgery Society. FBC = full blood count. LAGB = laparoscopic adjustable gastric banding. LFTs = liver function tests. PTH = parathyroid hormone. S = measure if concerning signs or symptoms. U&Es = urea and electrolytes.*

Both NICE and EASO suggest that long-term care is delivered in primary care.^3^^,^^5^ However, there are no specific healthcare funding or services available to support GPs to undertake long-term care annual reviews and there are concerns that patients are not being reviewed, resulting in risk of avoidable harms and outcomes not being optimised.^3^^,^^5^

To date, there has been no research into the long-term routine care and monitoring of patients after bariatric surgery in primary care. The aim of this study was to investigate whether the nutritional care and weight monitoring delivered by GPs to patients 2 years post-bariatric surgery meets current UK national clinical guidance.

## METHOD

### Study design

A retrospective cohort study of patients who have had bariatric surgery was conducted using routinely collected primary care data, starting follow-up from the second year post-surgery (when care is transferred back to primary care) to estimate the annual proportion of patients with a record of weight measurement, measurement of nutritional screening blood tests recommended by BOMSS guidelines,^20^ and prescription of nutritional supplements recommended by BOMSS guidelines.^20^ A secondary aim was to examine the proportion of patients whose test result indicated a nutritional deficiency.

### Data source

The IQVIA Medical Research Data (IMRD) UK database is an electronic primary care database, which contains pseudoanonymised electronic medical records of patients from 787 general practices. It provides longitudinal patient records of more than 15 million patients and covers around 6.2% of the UK population (https://www.the-health-improvement-network.com/en/). IMRD is generalisable to the UK population, including medical records of patients from all ages, sexes, and socioeconomic groups.^21^ It has previously been validated for the purpose of studying chronic conditions such as obesity and type 2 diabetes mellitus.^22^

### Study population

The study population was extracted from GP practices that had met the inclusion criteria of using the Vision electronic medical record system for at least 1 year and showing Acceptable Mortality Recording for at least 1 year before being considered for data extraction. From the eligible GP practices, cohort entry was restricted to adult patients (≥18 years) with a body mass index (BMI) ≥30 kg/m^2^ before surgery and a Read code record of a bariatric surgery procedure in their medical records at any time between 1 January 2000 and 1 January 2015 (see Supplementary Table S1 for details of Read codes). This study focused on patients who had been discharged from specialist care at 2 years post-surgery, therefore patients needed to have had a minimum of 3 years follow-up since surgery for inclusion.

The study focused on the bariatric procedures most commonly conducted in the UK: laparoscopic adjustable gastric banding (LAGB), gastric bypass, and sleeve gastrectomy. To be eligible for inclusion, study participants must have been registered with their practice for at least 1 year before study entry to ascertain documentation of concomitant diseases and treatments. Patients needed to have a BMI ≥30 kg/m^2^ to minimise the inclusion of patients who might have had bariatric surgery for reasons other than obesity.

### Outcomes

Estimations were made of the annual proportion of patients in the third, fourth, and fifth year of follow-up post-surgery for whom nutritional screening blood tests were requested as recommended by BOMSS guidelines, a measurement of weight/BMI was recorded, and records for prescriptions of nutritional supplements recommended by BOMSS were available. Box 1 summarises BOMSS nutritional guidance for each bariatric procedure.^20^ Study follow-up was from the index date (2 years post-bariatric surgery) until the earliest of the following end points: death date, date patient left the practice, date practice ceased to contribute to the database, and study end date (17 January 2018).

The nutritional screening blood tests recommended by BOMSS^20^ were defined by Read codes (see Supplementary Table S2 for details) or based on the availability of blood test measurements. To summarise the results as concisely as possible, creatinine level was used as a proxy for measurement of urea and electrolytes (U&Es) as serum levels are usually only measured as part of the panel of tests included in U&Es. Similarly, protein was used as a proxy for measurement of liver function tests. Protein was chosen because it is a clinically important measurement for patients postbariatric surgery due to the risks of protein malnutrition.^23^ Haemoglobin (Hb) was used as a proxy for measurement of full blood count (FBC) as it is usually only measured as part of the panel of tests in FBC. Prescriptions of nutritional supplements recommended by BOMSS nutritional guidance were defined by drug codes (see Supplementary Table S3 for details). Prescriptions were included for all possible relevant nutritional supplements as listed in the *British National Formulary*.^24^ For those patients who had a nutritional screening blood test, the proportion whose test result indicated nutritional deficiency was estimated. Nutrient levels that indicated a deficiency were based on laboratory levels used in the Tier 3/4 bariatric services across University Hospitals Birmingham NHS Foundation Trust.

### Analysis

Descriptive analysis of the baseline characteristics was performed and expressed as mean (standard deviation [SD]) or frequency (%) depending on whether the variable was continuous or categorical.

The annual proportion of patients who received nutritional blood test screening, weight screening, or nutritional supplement prescriptions was estimated. The proportion of patients who had had a nutritional screening blood test with a nutritional deficiency was also estimated. Compliance with recommended nutritional and weight monitoring, and nutritional supplement prescriptions, was analysed by conducting sequential analysis for serial 12-month periods starting from 2 years post-surgery. When estimating screening compliance in years 2–3, 3–4, and 4–5, patients were restricted to those with a minimum follow-up post-surgery of 3, 4, and 5 years, respectively. Therefore, for example, for year 3 compliance estimation, the denominator was patients who underwent bariatric surgery and were followed up in the IMRD database until 3 years post-surgery. The numerator was the number of those patients with a record of a given screening test/nutritional prescription/weight measurement from Read codes/test results/drug codes between year 2 and 3 post-bariatric surgery. This was repeated for years 4 and 5. Annual proportions were also estimated stratified by the type of surgical procedure because guidance varies with surgical procedure. A Cochran–Armitage test was used to assess whether any observed temporal trends in annual proportions were statistically significant. Stata (version 15) statistical software was used for data analysis.

## RESULTS

After excluding patients with a BMI <30 kg/m^2^ before surgery (*n* = 186), 3137 patients with a Read code record of a bariatric surgery procedure and a minimum follow-up of 3 years post-surgery were eligible for inclusion. Of these patients, 1400 (44.6%) had a Read code for LAGB, 1067 (34.0%) for gastric bypass, 446 (14.3%) for sleeve gastrectomy, and 224 (7.1%) patients had a record of other bariatric surgery procedures (Table 1). Twenty per cent of the cohort were male (*n* = 633) and mean age at surgery was 48.4 years (SD 10.3). The mean BMI pre-surgery was 45.3 kg/m^2^ (SD 8.9) and mean BMI post-surgery was 36.8 kg/m^2^ (SD 8.8). Just under a fifth (19.5%) of the cohort (*n* = 610) were in the most affluent Townsend deprivation quintile. Most patients (with a record of ethnicity) were of white ethnicity (52%, *n* = 1637), with only very small numbers from other ethnicities. Baseline characteristics were similar for patients having different bariatric procedures (Table 1). Median follow-up post-surgery was 5.7 years (interquartile range 4.2–7.6).

**Table 1.** Baseline characteristics of patients

**Variable**	**Total (*n*= 3137)**	**LAGB (*n*= 1400)**	**Gastric bypass(*n*= 1067)**	**Sleeve gastrectomy(*n*= 446)**
Age at the time of surgery, mean years (SD)	48.4 (10.3)	47.3 (9.9)	48.8 (10.3)	50.6 (10.6)

Male sex, *n* (%)	633 (20.2)	206 (14.7)	246 (23.1)	120 (26.9)

**Number of patients with available BMI pre-surgery data, *n* (%)**	3076 (98.1)	1373 (98.1)	1050 (98.4)	437 (98.0)
BMI pre-surgery, mean (SD)^a^	45.3 (8.9)	43.3 (8.5)	46.8 (7.9)	47.6 (9.1)
BMI pre-surgery, median (IQR)^a^	44.6 (39.3 to 50.2)	42.3 (37.9 to 47.3)	46.7 (41.4 to 51.5)	46.8 (41.7 to 52.8)

**Number of patients with available BMI post-surgery data, *n* (%)**	2097 (66.9)	1031 (73.6)	680 (63.7)	245 (54.9)
BMI post-surgery, mean (SD)^a,b^	36.8 (8.8)	37.2 (8.8)	34.9 (7.9)	38.3 (8.5)
BMI post-surgery, median (IQR)^a,b^	36.1 (30.7 to 41.7)	36.3 (30.9 to 42.3)	34.2 (29.5 to 39.1)	38.2 (32.3 to 43.5)
Year of last available BMI recording post-surgery, mean (SD)^a^	2.7 (2.1)	3.3 (2.3)	2.1 (1.7)	1.9 (1.6)
Year of last available BMI recording post-surgery, median (SD)^a^	2.3 (1.0 to 4.0)	3.0 (1.5 to 4.7)	1.8 (0.7 to 3.2)	1.5 (0.7 to 2.4)

**Townsend deprivation quintile, *n* (%)**				
1	610 (19.5)	312 (22.3)	190 (17.8)	73 (16.4)
2	526 (16.8)	251 (17.9)	170 (15.9)	66 (14.8)
3	594 (18.9)	280 (20.0)	196 (18.4)	79 (17.7)
4	559 (17.8)	213 (15.2)	214 (20.1)	88 (19.7)
5	405 (12.9)	157 (11.2)	151 (14.2)	61 (13.7)
Missing data	443 (14.1)	187 (13.4)	146 (13.7)	79 (17.7)

**Ethnicity, *n* (%)**				
White	1637 (52.2)	692 (49.4)	585 (54.8)	244 (54.7)
African-Caribbean	61 (1.9)	19 (1.4)	29 (2.7)	11 (2.5)
South Asian	48 (1.5)	14 (1.0)	24 (2.3)	6 (1.4)
Mixed race	11 (0.4)	7 (0.5)	3 (0.3)	0 (0.0)
Chinese/Middle Eastern/Other	14 (0.5)	10 (0.7)	2 (0.2)	2 (0.5)
Missing data	1366 (43.5)	658 (47.0)	424 (39.7)	183 (41.0)

a *Summary statistics based on available data only.*

b *Last recording available in the database. BMI = body mass index. IQR = interquartile range. LAGB = laparoscopic adjustable gastric banding. SD= standard deviation.*

### Weight measurements

**Figure 1.**
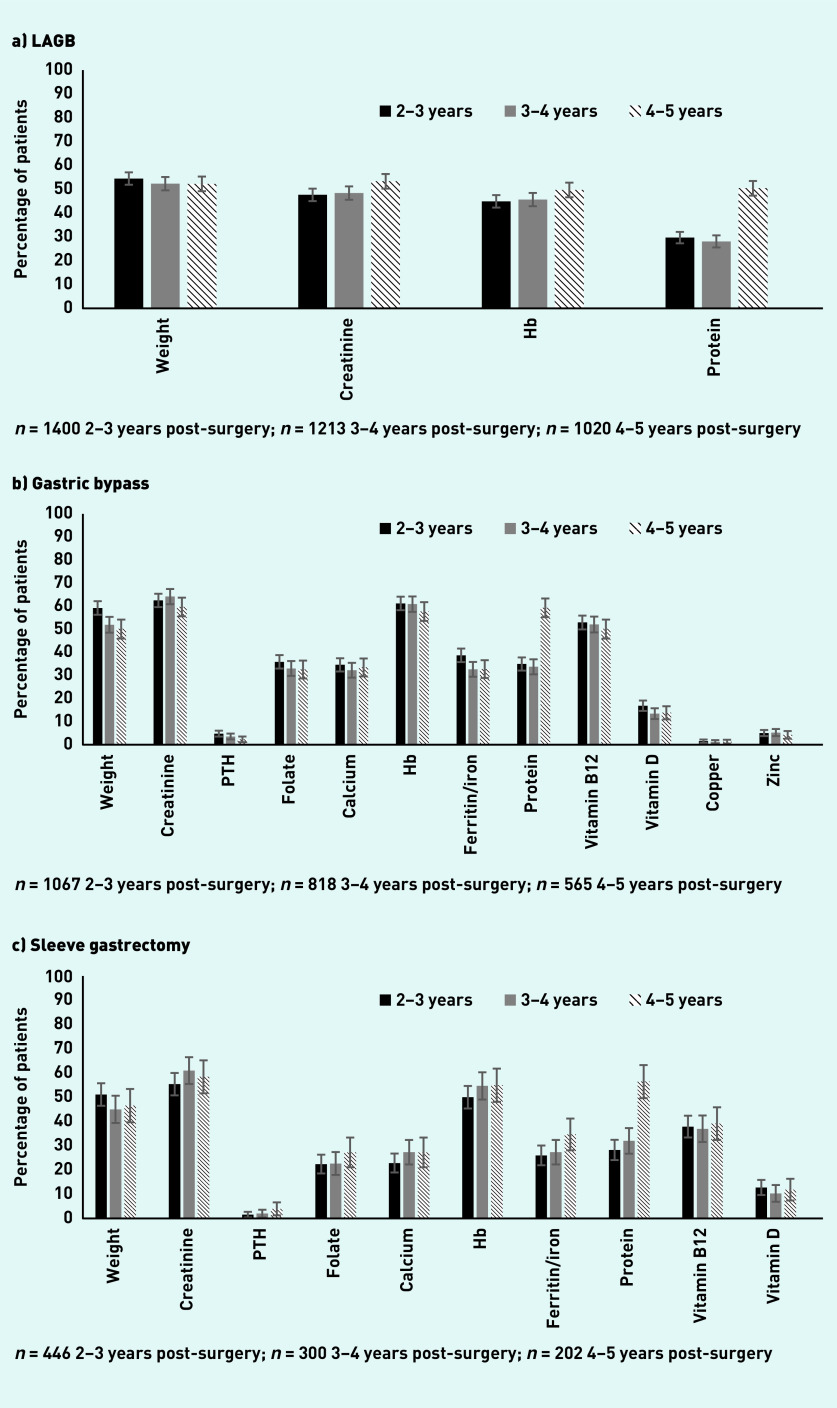
***Records of weight measurements and blood tests.****^a^* *^a^****Error bars are 95% confidence intervals. Hb = haemoglobin. LAGB = laparascopic adjustable gastric banding. PTH = parathyroid hormone.***

Table 2 gives the records of weight measurements for patients at 2–3 years, 3–4 years, and 4–5 years post-surgery. A total of 54.5% of patients (*n* = 763) who had had a LAGB had a weight recorded in year 2–3 post-surgery (the first year following specialist discharge). This remained steady in years 3–4 and 4–5 post-surgery (*P* = 0.250 for temporal trend) (Figure 1a).

**Table 2.** Records of blood tests and weight measurements

**Variable**	**2–3 years post-surgery**			**3–4 years post-surgery**			**4–5 years post-surgery**		
	**LAGB (*n*= 1400) *n* (%)**	**Gastric bypass (*n*= 1067) *n* (%)**	**Sleeve gastrectomy (*n*= 446) *n* (%)**	**LAGB (*n*= 1213) *n* (%)**	**Gastric bypass (*n*= 818) *n* (%)**	**Sleeve gastrectomy (*n*= 300) *n* (%)**	**LAGB (*n*= 1020) *n* (%)**	**Gastric bypass (*n*= 565) *n* (%)**	**Sleeve gastrectomy (*n*= 202) *n* (%)**
**Weight**	763 (54.5)	632 (59.2)	228 (51.1)	635 (52.4)	425 (52.0)	135 (45.0)	533 (52.3)	283 (50.1)	94 (46.5)
**Blood tests recommended by BOMSS for all patients**									
**Creatinine**	667 (47.6)	667 (62.5)	247 (55.4)	587 (48.4)	525 (64.2)	183 (61.0)	544 (53.3)	337 (59.7)	118 (58.4)
**Albumin**	607 (43.4)	624 (58.5)	216 (48.4)	519 (42.8)	490 (59.9)	160 (53.3)	486 (47.7)	314 (55.6)	98 (48.5)
**Parathyroidhormone**	6 (0.4)	51 (4.8)	7 (1.6)	6 (0.5)	29 (3.6)	6 (2.0)	5 (0.5)	13 (2.3)	8 (4.0)
**Folate**	215 (15.4)	383 (35.9)	100 (22.4)	175 (14.4)	270 (33.0)	68 (22.7)	171 (16.8)	184 (32.6)	55 (27.2)
**Calcium**	291 (20.8)	369 (34.6)	102 (22.9)	236 (19.5)	264 (32.3)	82 (27.3)	223 (21.9)	189 (33.5)	55 (27.2)
**Haemoglobin**	629 (44.9)	653 (61.2)	223 (50.0)	554 (45.7)	498 (60.9)	164 (54.7)	507 (49.7)	326 (57.7)	111 (55.0)
**Ferritin/iron**	222 (15.9)	413 (38.7)	116 (26.0)	193 (15.9)	267 (32.6)	82 (27.3)	197 (19.3)	185 (32.7)	70 (34.7)
**Protein**	416 (29.7)	373 (35.0)	126 (28.3)	341 (28.1)	276 (33.7)	96 (32.0)	514 (50.4)	335 (59.3)	114 (56.4)
**Vitamin B12**	242 (17.3)	565 (53.0)	169 (37.9)	199 (16.4)	426 (52.1)	111 (37.0)	194 (19.0)	283 (50.1)	79 (39.1)
**Vitamin D**	65 (4.6)	180 (16.9)	57 (12.8)	56 (4.6)	110 (13.5)	31 (10.3)	59 (5.8)	78 (13.8)	24 (11.9)
**Copper**	1 (0.07)	16 (1.5)	4 (0.9)	6 (0.5)	10 (1.2)	2 (0.7)	1 (0.1)	7 (1.2)	3 (1.5)
**Zinc**	14 (1.0)	54 (5.1)	11 (2.5)	17 (1.4)	43 (5.3)	9 (3.0)	8 (0.8)	24 (4.3)	5 (2.5)
**Blood tests recommended by BOMSS depending on symptoms and diagnoses**									
**Vitamin E**	0 (0.0)	1 (0.1)	0 (0.0)	2 (0.2)	2 (0.2)	1 (0.3)	0 (0.0)	1 (0.2)	1 (0.5)
**Vitamin K**	0 (0.0)	0 (0.0)	0 (0.0)	0 (0.0)	0 (0.0)	0 (0.0)	0 (0.0)	0 (0.0)	0 (0.0)
**Vitamin A**	2 (0.1)	3 (0.3)	2 (0.5)	3 (0.3)	5 (0.6)	2 (0.7)	0 (0.0)	5 (0.9)	4 (2.0)
**Magnesium**	26 (1.9)	53 (5.0)	12 (2.7)	21 (1.7)	38 (4.7)	11 (3.7)	18 (1.8)	33 (5.8)	4 (2.0)
**Selenium**	3 (0.2)	5 (0.5)	2 (0.5)	3 (0.3)	11 (1.3)	3 (1.0)	3 (0.3)	7 (1.2)	3 (1.5)
**Thiamine**	0 (0.0)	1 (0.1)	2 (0.5)	0 (0.0)	1 (0.1)	2 (0.7)	0 (0.0)	0 (0.0)	1 (0.5)

*BOMSS = British Obesity and Metabolic Surgery Society. LAGB = laparoscopic adjustable gastric banding.*

Of patients who had had a gastric bypass, 59.2% (*n* = 632) had a record of a weight in year 2–3 post-surgery. This fell to 52.0% (*n* = 425) at year 3–4 post-surgery and to 50.1% (*n* = 283) at year 4–5 post-surgery (*P* = 0.001 for temporal trend) (Figure 1b).

Of patients who had had a sleeve gastrectomy, 51.1% (*n* = 228) had a recorded weight measurement in year 2–3 post-surgery, 45.0% (*n* = 135) at year 3–4, and 46.5% (*n* = 94) at year 4–5 (*P* = 0.176 for temporal trend) (Figure 1c).

### Nutritional monitoring blood tests

Table 2 lists the blood tests that were recorded for patients at 2–3 years, 3–4 years, and 4–5 years post-surgery. Records of a measurement of nutritional monitoring blood tests recommended for LAGB varied between 29.7% for protein (*n* = 416) and 47.6% for creatinine (*n* = 667) in year 2–3 post-surgery, while 44.9% (*n* = 629) had a record of Hb measured in year 2–3 post-surgery (Figure 1a). These annual proportions were similar in year 3–4 post-surgery, with a small increase in the proportions of patients with a record of Hb or creatinine measurement, and a larger increase in the proportion with a record of a protein measurement in year 4–5 (*P* = 0.024, *P* = 0.008, and *P*<0.001 for Hb, creatinine, and protein temporal trends, respectively) (Figure 1a).

For both gastric bypass and sleeve gastrectomy, there was a marked difference in the annual proportions of patients with a record of a measurement of a routinely requested blood test (such as Hb and creatinine) and the proportions with a record of a measurement of a blood test more specific to bariatric surgery (Figures 1b and 1c). For example, 59.7–64.2% of patients who had a gastric bypass had a record of creatinine measurement but only 4.3–5.3% had measurements for zinc and only 1.2–1.5% had measurements for copper (Figure 1b).

### Symptom- or diagnosis-dependent blood tests

Annual proportions of patients with a record of one of the blood tests recommended depending on patient symptoms were all very low, with several (for example, vitamins A, E, K, and selenium) recorded for <1% of patients (Table 2).

### Nutritional deficiencies

Where results were available, records indicated that the most common deficiencies were low haemoglobin, which varied between 40.5% (sleeve gastrectomy) and 50.6% (gastric bypass and LAGB) of patients, and low ferritin levels, which varied between 18.9% (LAGB) and 35.0% (gastric bypass and LAGB). The full results of records indicating a nutritional deficiency are outlined in Table 3.

**Table 3.** Records of a result indicating a deficiency

**Variable**	**2–3 years post-surgery**			**3–4 years post-surgery**			**4–5 years post-surgery**		
	**LAGB *n*/*N*^a^ (%)**	**Gastric bypass *n*/*N* (%)**	**Sleeve gastrectomy *n*/*N* (%)**	**LAGB *n*/*N* (%)**	**Gastric bypass *n*/*N* (%)**	**Sleeve gastrectomy *n*/*N* (%)**	**LAGB *n*/*N* (%)**	**Gastric bypass *n*/*N* (%)**	**Sleeve gastrectomy *n*/*N* (%)**
Folate (AHD <3.1 microgram/L)	15/192 (7.8)	12/361 (3.3)	7/98 (7.1)	11/158 (6.9)	13/257 (5.1)	6/67 (9.0)	16/157 (10.2)	3/173 (1.7)	2/55 (3.6)
Calcium (AHD <2.2 mmol/L)	22/262 (8.4)	53/325 (16.3)	11/92 (12.0)	25/213 (11.7)	33/238 (13.9)	6/77 (7.8)	23/200 (11.5)	26/172 (15.1)	7/51 (13.7)
Anaemia (AHD <133 (male)/110 (female) g/L)	283/623 (45.4)	327/646 (50.6)	89/220 (40.5)	277/548 (50.6)	247/496 (50.0)	75/162 (46.3)	233/498 (46.8)	162/325 (49.9)	46/110 (41.8)
Ferritin (AHD <15 microgram/L)	32/169 (18.9)	89/347 (25.7)	20/95 (21.1)	48/137 (35.0)	69/224 (30.8)	22/72 (30.6)	43/153 (28.1)	57/163 (35.0)	18/61 (29.5)
Protein (AHD <60 g/L)	7/415 (1.7)	11/372 (3.0)	2/125 (1.6)	5/339 (1.5)	6/276 (2.2)	1/96 (1.0)	4/320 (1.3)	7/188 (3.7)	0/61 (0.0)
Vitamin B12 (AHD <187 ng/L)	22/190 (11.6)	52/360 (14.4)	14/99 (14.1)	20/154 (13.0)	40/280 (14.3)	6/68 (8.8)	16/156 (10.3)	25/186 (13.4)	4/52 (7.7)
Parathyroid hormone (AHD >7.2 pmol/L)	1/6 (16.7)	15/51 (29.4)	0/7 (0.0)	1/6 (16.7)	11/29 (37.9)	3/6 (50.0)	2/5 (40.0)	7/13 (53.9)	2/8 (25.0)

a N *is the total number of patients with a record of a given nutritional blood test measurement.* n *is the number of patients with a record of a given nutritional blood test measurement for whom the result indicated a deficiency in that nutrient. Only those with a recorded blood test result were included in these analyses AHD = additional health data.*

*LAGB = laparoscopic adjustable gastric banding.*

### Prescription of nutritional supplements

**Figure 2.**
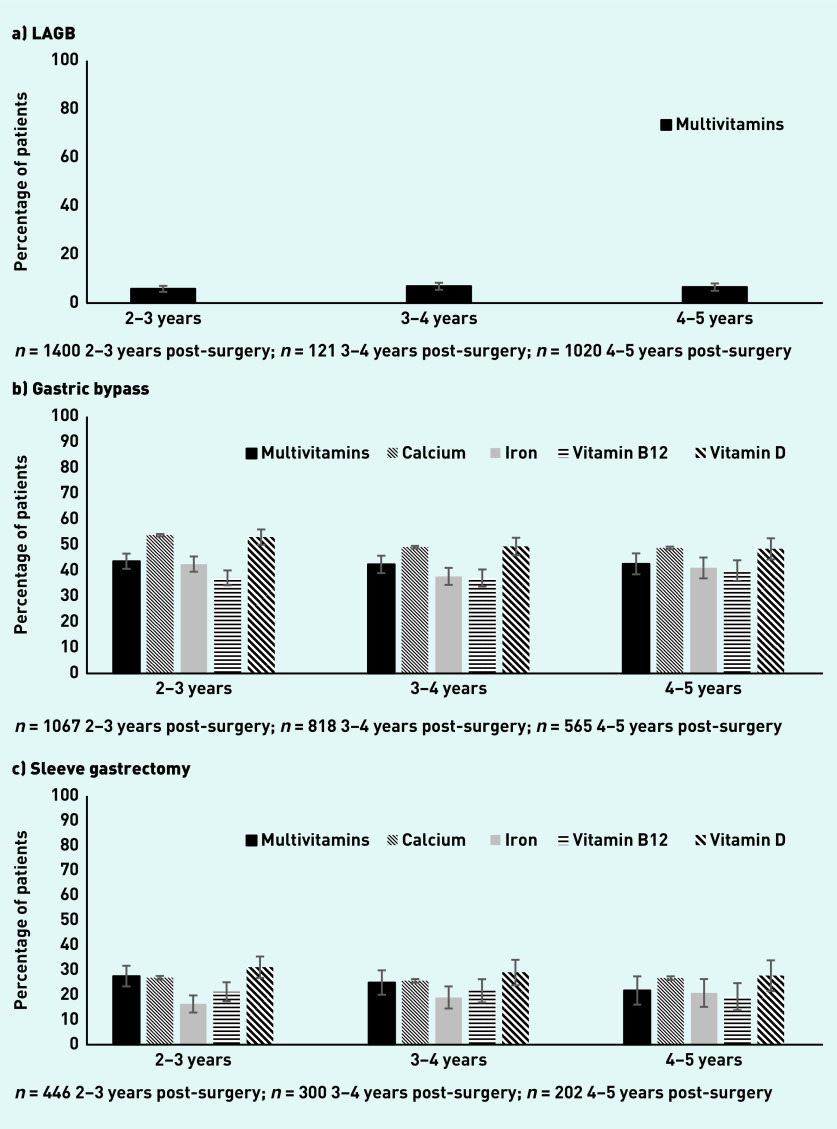
***Records of a prescription of recommended nutritional supplements.****^a^* *^a^****Error bars are 95% confidence intervals. LAGB = laparoscopic adjustable gastric banding.***

Only 5.9–6.9% of patients who had had a LAGB had a record of a prescription for a multivitamin prescription in each given year (Figure 2a).

For gastric bypass, the annual proportion of patients with a record of a multivitamin prescription was 42.4–43.7%, while the annual proportion with a record of a prescription for iron was 37.8–42.6% and for vitamin B12 was 37.2–40.0% (Figure 2b). The annual proportion with a record of a prescription for folic acid varied between 10.0% and 10.4% and for calcium/vitamin D varied between 48.5% and 53.8% (Figure 2b).

The annual proportion of patients who had had a sleeve gastrectomy with a record of a prescription for each of the supplements were all lower than those who had undergone a gastric bypass, varying between 8.3% for folic acid (year 2–3) and 31.2% for vitamin D (year 2–3) (Figure 2c).

Annual proportions of supplement prescriptions for all the procedures did not vary appreciably with time (*P*>0.05 for trend over time), except for a decrease in the proportion of calcium prescriptions among patients who underwent bypass surgery (*P* = 0.034 for trend).

## DISCUSSION

### Summary

These results suggest that patients are not receiving the long-term nutritional care recommended in national guidance. There was a marked contrast between the proportion of patients having routine blood tests and the very low proportion having blood tests more specific to bariatric surgery follow-up. It is possible that these more specific blood tests are a truer reflection of the incidence of post-bariatric surgery annual nutritional reviews since tests routinely carried out in primary care could be requested for a multitude of reasons other than bariatric surgery follow-up. If results for the more specific tests are used as a proxy for an annual bariatric surgery review, it would suggest that only around 5% of patients are receiving recommended long-term follow-up reviews in primary care.

### Strengths and limitations

To the authors’ knowledge, this is the first study to investigate the care patients receive in primary care post-bariatric surgery after specialist discharge and whether it meets current clinical guidelines. The IMRD database enabled the use of routinely collected data that included a large number of patients with good national coverage over 3 years follow-up in primary care. These data should be representative of the current routine clinical care received by patients. However, it was not possible to obtain data about the indications for blood tests or supplement prescriptions, so it is not known if they were requested or prescribed for reasons unrelated to bariatric surgery. Whether the correct dose of a given nutritional supplement was being prescribed was not investigated, only whether a prescription had been issued. It is possible that some nutritional supplements are obtained over the counter or from specialist services so the data may underestimate supplement use. However, generally specialist bariatric services are not commissioned for long-term followup and it is likely that only very small numbers of patients would receive long-term supplements via these services. Read codes for bariatric surgery may have included patients having bariatric surgery for reasons other than obesity, such as stomach cancer. However, a feasibility check suggested that such patients represented <1% of patients in the study.

### Comparison with existing literature

Previous studies have shown that adherence to follow-up care and nutritional supplements can be poor, leading to an increased risk of nutritional deficiencies and weight regain.^16^^–^^18^^,^^25^ The levels of deficiencies reported in these previous studies were generally lower than those reported in the current study.^17^ There may be many reasons for this, including differences in study population and study design.

There has been little previous research about the long-term care patients receive in primary care following discharge from specialist follow-up. In 2019 Mahawar *et al*^26^ conducted a survey of UK adult patients who had had bariatric surgery regarding adherence to nutritional supplements. They reported that, as well as patients forgetting to take medication, GPs not prescribing supplements was a barrier, and recommended that patient and GP education may help.^26^ Several survey studies have reported a lack of confidence among GPs in managing patients who have undergone bariatric surgery, as well as a desire for more education.^27^^,^^28^ This suggests that GP confidence and education may be barriers to patients receiving long-term care post-bariatric surgery. There have been some attempts to improve GPs’ awareness of the management of patients following bariatric surgery in primary care in the UK through the development of guidance specifically for GPs.^29^^,^^30^ However, any impact these resources may have had is not clear.

### Implications for research and practice

International clinical consensus is that long-term follow-up care following bariatric surgery is important to optimise patient outcomes and reduce the risk of preventable harms.^3^^,^^5^^,^^8^^–^^10^ This study suggests that patients are not receiving the recommended nutritional care post-specialist discharge in terms of monitoring and treatment, increasing the risk of preventable adverse outcomes. The importance of appropriate follow-up post-bariatric surgery should be emphasised to healthcare professionals and patients, and GPs supported to provide this care.

Future research should aim to understand the reasons underpinning the apparent lack of follow-up to help to develop appropriate strategies to improve the care of patients post-bariatric surgery.
